# Annotations

**Published:** 1908-07-11

**Authors:** 


### ANNOTATIONS.

### "Common Law and Common Sense."

A LONDON evening paper happily sums up thus the decision of Justice Channell on July 6 in the King's Bench Division over a very knotty point connected with accident insurance. The insured was hunting when his horse touched a strand of barbed wire concealed in a fence, and threw him off on to wet ground. Thus soaked through, he at once rode home at a trot; but this took an hour and a half. It was found that to ride home thus was the course least likely to aggravate the effects of the accident in the circumstances; and that the fall, wetting, and exposure in combination were the cause of a pneumonia which supervened the next day, by diminishing the general vitality of the system and the local resistance of the lung so that pneumococci were enabled to multiply in the latter. The insurance company relied on a clause in the policy excluding "disease or other intervening cause" as the "direct or proximate cause" of death. The case hinged on the exact meaning of "direct or proximate." The company held that death was due to pneumonia; the claimant, to accident and exposure: and with some doubt the learned judge decided in favour of the latter. Probably this decision, besides being common sense, is really in the interests of the companies themselves, for if their contention had been upheld, it is reasonable to suppose that many people would refuse to insure on such policies. The same principle is observed in dealing with disputes under the Workmen's Compensation Acts: thus an award has been made for tuberculosis of the hip developing after a severe sprain in that joint caused by the snapping of a crowbar used by the workman. So, too, about a fortnight ago, an obviously correct verdict of suicide was returned by a coroner's jury in a case of death from pneumonia following self-immersion. The line between accident and disease is admittedly very difficult to draw, but in this case common law, common sense, and common policy seem to be all upon the same side of it.

### Graduated Exercises in Phthisis.

A LARGE party of visitors travelled to the Brompton Hospital Sanatorium at Frimley on July 4 to inspect the premises and grounds, and to observe in actual operation the method of treatment adopted in the institution since its opening four years ago. The characteristic of the sanatorium is that the patients, both male and female, are systematically employed on such light work as they are capable of performing. Under this system of graduated labour the patients perform harder and harder tasks as they regain health, until, in the case of the men, they are able to do the hardest navvy's work. There are seven grades of labour and exercise. Patients who are unfitted for active exercise are given light manual employment. When their condition is sufficiently improved they walk from one to six miles a day, and then they are set to progressively heavier tasks. The patients, unaided by paid labour, keep the whole of the grounds and gardens in order; and in addition, since the opening of the sanatorium, they have effected a number of permanent improvements. Their latest work is the construction of a concrete reservoir, now nearly finished, to hold half a million gallons of water. The full complement of the sanatorium is at present 110 patients. Only such cases as seem suitable for open-air treatment are received, and those admitted are mostly chosen from among the out-patients of the Brompton Hospital, in the wards of which they are kept under observation until it has been determined that they are fit to be sent to Frimley. The average length of stay in the sanatorium is from four to six months. The annual cost per bed, including all maintenance charges, is about £65. Patients are not discharged until it is certified that the disease has been arrested. It is satisfactory to note that of those who have left the institution and returned to their former occupations, at least 80 per cent. are still in full work. Funds are urgently needed to maintain the sanatorium, the annual requirement being £7,000.

### Australia and Quack Medicines.

WE read lately in the *Pall Mall Gazette* that Mr. Henry D. Baker, a United States Consul in Tasmania, has issued a warning to patent medicine proprietors whose works are situated in the United States that they incur risk of large losses if they export goods to Australia accompanied by labels or wrapped with circulars making claims as to their curative properties which cannot be substantiated. Mr. Baker makes reference to a large shipment of patent medicines detained at the Customs House, of which the Collector of Customs has written to say that they "are detained on account of extravagant claims made as to their curative properties. The statements on the cartons, labels, and so forth are extremely misleading." The Consul says he examined one of the wrappers in the shipment in question, and counted forty different maladies for which the medicine was alleged to be a specific. Had the medicine been a cure for thirty-nine less maladies, he observes, it would have had a better chance to get through the Custom House. We rejoice to hear that Australia has already commenced operations in the campaign which she has undertaken against quackery and commercial fraud. It is a matter for regret that England should lag behind one of her colonies in this matter. But public opinion in this country is still grotesquely tolerant of extravagant claims of every sort, and apparently it must be educated further before legislative measures are instituted against the patent-medicine proprietor and his wares. In the meantime we shall watch with interest the advance of Australia, and shall hope that good will come of the deliberations of the Unqualified Practice Prevention Committee of the General Medical Council.

